# ﻿*Prunuszhuxiensis* (Rosaceae), a new species from Hubei, China

**DOI:** 10.3897/phytokeys.255.142428

**Published:** 2025-04-24

**Authors:** Qi-liang Gan, Wen-bin Xu, Xin-wei Li

**Affiliations:** 1 College of Pharmacy, Hubei University of Chinese Medicine, Wuhan 430065, Hubei, China Hubei University of Chinese Medicine Wuhan China; 2 Wuhan Botanical Garden, the Chinese Academy of Sciences, Wuhan 430074, Hubei, China Wuhan Botanical Garden, the Chinese Academy of Sciences Wuhan China

**Keywords:** China, Hubei, *Prunuszhuxiensis*, taxonomy

## Abstract

In the present paper, we describe a new species, *Prunuszhuxiensis* (P.subg.Cerasus), from Hubei, China, based on long-term field observations. This species closely resembles *P.serrulata* in having corymbose-racemose or subumbellate inflorescences, hairy petiole, pedicel, involucral bracts and black drupes. However, *P.zhuxiensis* differs distinctly from *P.serrulata* by its sweet edible drupes (versus bitter, inedible drupes in *P.serrulata*), stipules 4-lobed at the base (versus linear stipules in *P.serrulata*), smaller bracts, shorter pedicels and styles pilose at the base (versus glabrous styles in *P.serrulata*). Furthermore, molecular phylogenetic analyses indicate that *P.zhuxiensis* and *P.serrulata* are placed in separate clades, supporting their distinction.

## ﻿Introduction

Shi et al. (2013) re-defined the infrageneric relationships of *Prunus* L., based on molecular phylogenetic analyses. Currently, about 65 species are recognized within P.subg.Cerasus (Mill.) A.Gray, of which 44 species occur in China (Su et al. 2021; Yi et al. 2024b). Some new species within this subgenus have been reported from China in recent years (e.g. Xu et al. (2022); Liang et al. (2023); Yi et al. (2024a)). However, the phylogeny of Prunussubg.Cerasus inferred from chloroplast and/or nuclear genomic data (Hodel et al. 2021; Shen et al. 2023; Su et al. 2023) only partially corresponds with previously proposed morphological sectional classifications (Yu and Li 1986; Wang 2014) and several cases have also been detected in other lineages of Rosaceae, including the tribe Maleae (Liu et al. 2019, 2020, 2022, 2023; Jin et al. 2023, 2024; Wang et al. 2024) and the tribe Potentilleae (Xue et al. 2023).

During a botanical expedition conducted a few years ago in Zhuxi, Hubei, China, we encountered an interesting species of Prunussubg.Cerasus. After careful and detailed observations, we concluded that it represents a species new to botanical sciences, which we formally describe herein.

## ﻿Materials and methods

We collected specimens and took pictures of the new species in Zhuxi, Hubei, China. The specimens of the new species were deposited at the Herbarium of Wuhan Botanical Garden, CAS (HIB) and PE (China National Herbarium). We also checked the specimens at HIB, PE and CVH (Chinese Virtual Herbarium, https://www.cvh.ac.cn/) of Prunussubg.Cerasus. We carried out morphological comparisons in the field and herbaria. The specimens were observed with dissecting microscopes.

The complete chloroplast genome of the new species *Prunuszhuxiensis* (GenBank accession number: PV208095) was assembled using Getorganelle 1.7.7.0 (Jin et al. 2020) with Illumina genomic DNA sequencing data. We also downloaded chloroplast genomes of other *Prunus* species and two outgroup species (*Malusdomestica* (Suckow) Borkh. and *Spiraeamartini* H.Lév.). Before constructing phylogenetic trees, the chloroplast genomes were aligned with MAFFT 7.520 (Rozewicki et al. 2019) and the alignment was trimmed with Gblocks (Talavera and Castresana 2007) in Phylosuit 1.2.3 (Zhang et al. 2020). The Maximum Likelihood (ML) phylogenetic trees were generated with IQ-TREE 2.2.0 (ultrafast bootstrap 10000 replicates) (Nguyen et al. 2015) in Phylosuit 1.2.3 (Zhang et al. 2020). The nucleotide substitution model was determined with ModelFinder 2.2.0 (Kalyaanamoorthy et al. 2017) in Phylosuit 1.2.3 (Zhang et al. 2020) and then Bayesian Inference (BI) was performed using MrBayes 3.2.7 (10,000,000 generations; Ronquist et al. (2012)).

## ﻿Taxonomic treatment

### ﻿ Prunus zhuxiensis

Taxon classificationPlantaeRosalesRosaceae

Q.L.Gan, W.B.Xu & X.W.Li sp. nov.

478E3C2A-5BFF-5B48-A12C-85C8645451C8

urn:lsid:ipni.org:names:77360580-1

Figs 1 , 2 , 3

#### Diagnosis.

*Prunuszhuxiensis* is similar to *P.serrulata* Lindl. (Li and Bartholomew 2003; Yi et al. 2024b) in its hairy pedicel and involucral bracts, corymbose-racemose or subumbellate inflorescences and black drupes, but the flowers of *P.zhuxiensis* appear before the leaves (at the same time as leaves in *P.serrulata*) and have reflexed sepals half as long as the hypanthium (spreading sepals up to as long as hypanthium in *P.serrulata*) and style pilose at the base (glabrous in *P.serrulata*), the fruits of *P.zhuxiensis* are sweet and edible, while those of *P.serrulata* bitter and inedible.

#### Type.

China • Hubei Province, Zhuxi County, Quanxi Town, Baguashan Forest Farm, Hengduanshan, 32°3'50"N, 109°39'25"E, alt. 780 m, 15 March 2023, *Q.L. Gan 23-1-1* (***holotype***: HIB [barcode 0342513!]; ***Isotypes***, PE [barcodes 02553525!, 02553526!]).

***Paratypes***: • ibidem, alt. 780 m, 15 March 2023, *Q.L. Gan 23-1-2* (HIB [barcode 0346560!]), *Q.L. Gan 23-1-3* (HIB [barcode 0346559!]), *Q.L. Gan 23-1-4* (HIB [barcode 0346563!]); • ibidem, alt. 750 m, 26 April 2023, *Q.L. Gan 23-2-1* (HIB [barcode 0346561!]), *Q.L. Gan 23-2-2* (HIB [barcode 0346562!]), *Q.L. Gan 23-2-3* (HIB [barcode 0346564!]).

#### Description.

Trees, deciduous, 8–12 m tall, bark grey, lenticels elliptic or long elliptic, sparsely transversely arranged. Young branchlets purple or green, densely grey pubescent. Leaf blades narrowly obovate, obovate or elliptic, 3–12 × 1.5–5.5 cm, base cuneate or rounded, apex caudate or acuminate, margin crenately serrate or biserrate, teeth minute, tipped with apical glands, adaxially glabrous, abaxially white pilose on veins. Secondary veins 8–12 on each side. Petiole 10–14 mm, pubescent, with two purple disciform glands at upper part. Stipules 4-lobed at base, lobes margin capitate gland tipped, laciniato-fimbriate. Flowers emerging before leaves. Involucral bracts ovate, oblong, 4–7 × 3.5–4.5 mm, densely pubescent adaxially. Inflorescences umbellate or corymbose-racemose, 3–5-flowered. Peduncles short or absent. Pedicel 7–12 mm, pubescent. Bracts greenish-white, obovate, 1.5–2.5 × 1.5–2.5 mm, conical glandular serrate at apex. Hypanthium tabulate or campanulate, 6–7 × 2–3 mm, pubescent abaxially. Sepals ovate-lanceolate, 2–3 × 0.8–1 mm, reflexed. Styles 12–13 mm, pilose at base. Petals ovate or narrowly ovate, white, 10–13 × 6–7 mm, bifid at apex. Stamens 38–44, filaments up to 12 mm. Drupes globose or ovoid, black, 8–9 mm long, sweet, edible. Endocarp flat ovoid, 7–8 mm long, smooth.

**Figure 1.**
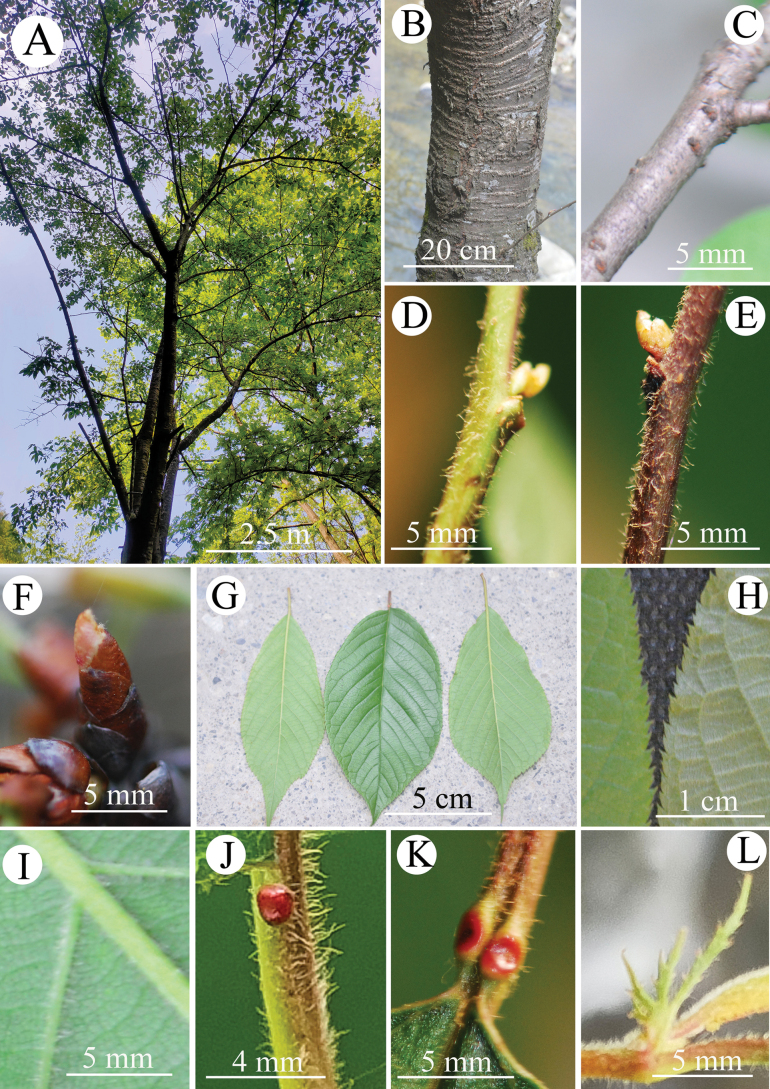
Vegetative characters of *P.zhuxiensis***A** crown **B** trunk **C** old branch **D** branchlet **E** branchlet **F** winter bud **G** leaf blades **H** teeth at leaf margin **I** hairs on lower surface of a leaf **J** hairs on a petiole **K** glands at apex of a petiole **L** stipule.

#### Phenology.

Flowering in March, fruiting in May.

#### Distribution and habitat.

*P.zhuxiensis* is distributed sparsely in the mixed evergreen and deciduous broad-leaved forest in the mountains or along streams at altitudes 600–1500 m around the type locality. The main accompanying species of *P.zhuxiensis* are *Salixwilsonii* Seemen ex Diels, *Sycopsissinensis* Oliv., *Camelliacuspidata* (Kochs) H.J.Veitch, *Phoebezhennan* S.K.Lee & F.N.Wei, *Juglansmandshurica* Maxim., *Albiziajulibrissin* Durazz., *Pterocaryastenoptera* C.DC., Cornuskousasubsp.chinensis (Osborn) Q.Y.Xiang, *Photiniabeauverdiana* C.K.Schneid etc.

**Figure 2.**
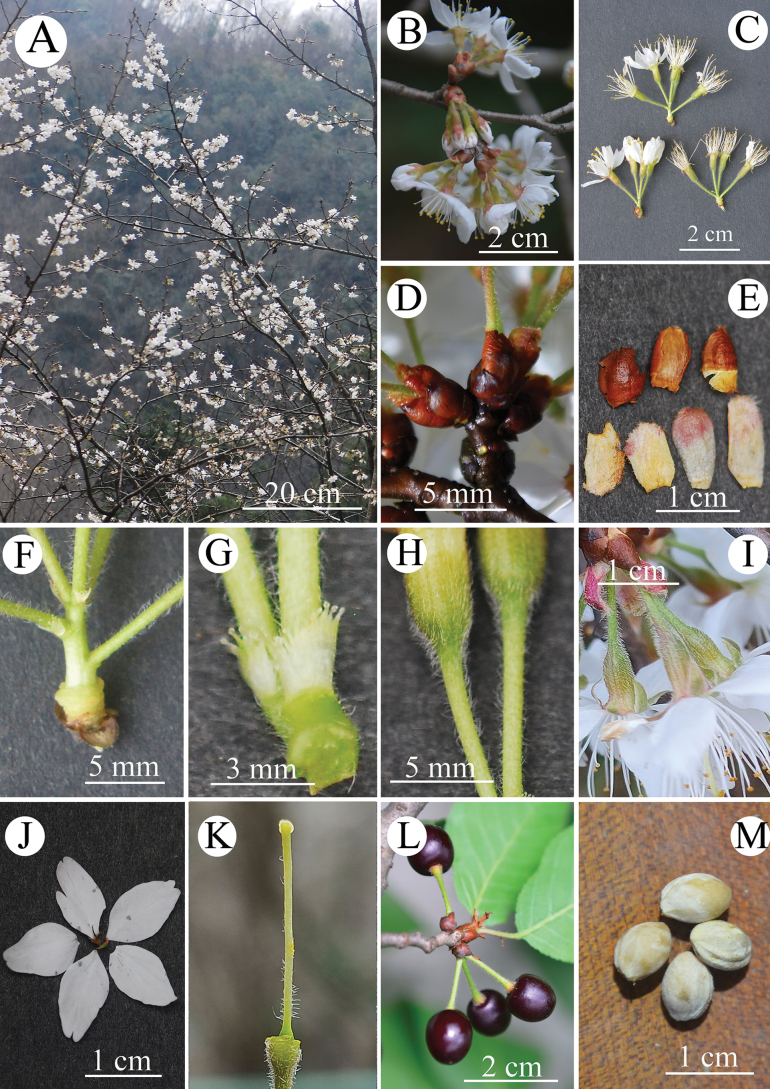
Reproductive characters of *P.zhuxiensis***A** flowering branches **B** inflorescences **C** inflorescences **D** involucre **E** involucral bracts **F** peduncle **G** bracts **H** hypanthium **I** hypanthium and bracts **J** petals **K** ovary and style **L** drupes **M** endocarps.

#### Etymology.

The specific epithet “*zhuxiensis*” refers to the type locality, Zhuxi, Hubei, China. The Chinese name of this species is Zhuxiyingtao (Pinyin).

#### Notes.

*P.zhuxiensis* co-occurs with *P.serrulata* in the same plant community. They are similar in their hairy pedicel and involucral bracts and black drupes. However, these two species can be distinguished by the pilose style base of *P.zhuxiensis* (which is glabrous in *P.serrulata*) and the sweet and edible drupes of *P.zhuxiensis* (compared to the bitter and inedible drupes of *P.serrulata*). The morphological differences are shown in Table 1, Suppl. material 1.

**Figure 3.**
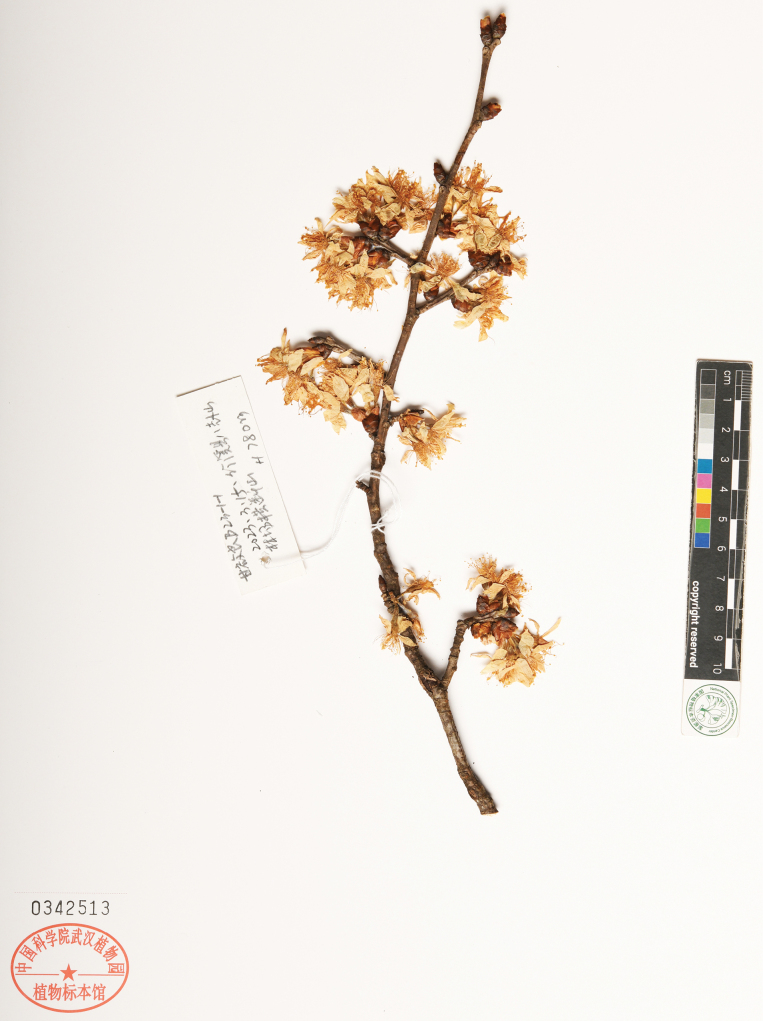
The holotype of *P.zhuxiensis*.

**Table 1.** Morphological comparison between *P.zhuxiensis* and *P.serrulata* (Li and Bartholomew 2003; Yi et al. 2024b).

Characters	*P.zhuxiensis*	*P.serrulata*
Petiole	apex with 2 disciform glands	apex with 1–3 rounded glands
Stipule	4-lobed at base	linear
Secondary veins	8–12 pairs	6–8 pairs
Phenology (in Zhuxi County)	flowering in March	flowering in April and May
Pedicel	0.7–1.2 cm	1.5–2.5 cm
Hypanthium	green or slightly purple	dark purple
Sepals	reflexed, 2–3 mm long, about half as long as hypanthium	spreading, 5 mm long, nearly as long as hypanthium
Petals	ovate or narrowly ovate	obovate
Style	pilose at base	glabrous
Bracts	greenish-white, 1.5–2.5 × 1.5–2.5 mm	brown or tinged greenish-brown, 5–8 × 2.5–4 mm
Fruits	black, sweet, edible	black, bitter, inedible

#### Molecular phylogeny.

**Figure 4.**
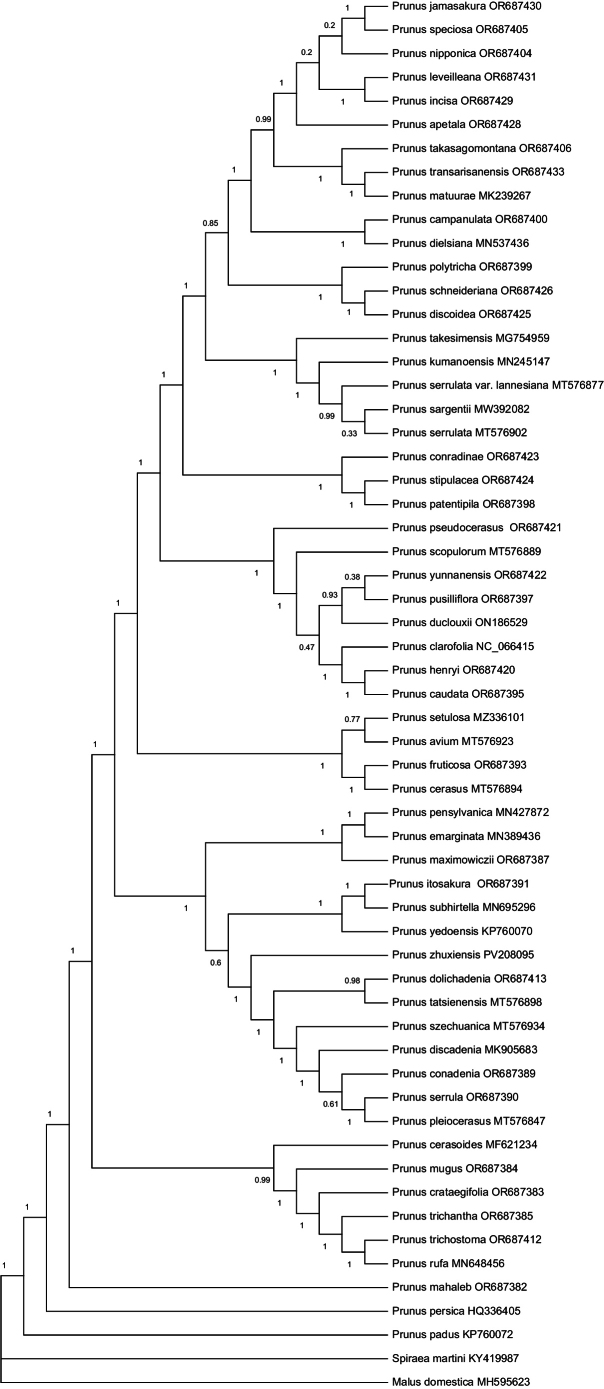
BI consensus tree of Prunussubg.Cerasus. GenBank accession number follows species name.

Both the BI and ML molecular trees (Fig. 4, Suppl. material 2) demonstrate the monophyly of Prunussubg.Cerasus, which is consistent with Shen et al. (2023). Shen et al. (2023) split the subg. Cerasus into seven lineages (Clades IIIa–IIIg) and the species of each of these lineages also cluster together in our phylogenetic trees. The new species *P.zhuxiensis* does not group with *P.serrulata* and it is nested in clade IIIc of Shen et al. (2023) and sister to a subclade consisting of *P.dolichadenia* Cardot, *P.tatsienensis* Batalin, *P.szechuanica* Batalin, *P.discadenia* Koehne, *P.conadenia* Koehne, *P.serrula* Franch. and *P.pleiocerasus* Koehne. *P.serrulata* and P.serrulatavar.lannesiana (Carrière) Makino and *P.sargentii* Rehder group together into a subclade as a part of clade IIIg of Shen et al. (2023). The present study shows that phylogenetic relatedness does not reflect the morphological resemblance of *P.zhuxiensis* and *P.serrulata*. While both *P.zhuxiensis* and *P.subhirtella* are placed in the same clade named IIIc of Shen et al. (2023), these two species share the characters of black drupes and hairy inflorescences and pilose style.

##### ﻿Key to species of Prunussubg.Cerasus in Zhuxi, Hubei, China (based on Li and Bartholomew (2003))

**Table:** 

1a	Bracts green, persistent	**2**
2a	Glands disciform or depressed at apex of teeth along bract margins	**3**
3a	Inflorescences subcorymbose-racemose or racemose	***Prunusszechuanica***
3b	Inflorescences umbellate	***Prunustatsienensis***
2b	Glands not disciform or depressed at apex of teeth along bract margins	**4**
4a	Bracts 5–20 mm; sepals spreading	***Prunussetulosa***
4b	Bracts 2–8 mm; sepals reflexed	**5**
5a	Hypanthium outside densely pilose	***Prunuswangii***
5b	Hypanthium outside glabrous	**6**
6a	Stamens 20–30; drupe long ellipsoid	***Prunusclarofolia***
6b	Stamens 32–54; drupe ovoid to subglobose	***Prunusconradinae***
1b	Bracts brown or rarely greenish-white, rarely persistent	**7**
7a	Inflorescences more or less hairy or at least hairy when young	**8**
8a	Style glabrous	**9**
9a	Sepals about 1/2 as long as hypanthium	***Prunuspseudocerasus***
9b	Sepals about 2 times as long as hypanthium	***Prunusdielsiana***
8b	Style hairy	**10**
10a	Hypanthium tubular, base dilated.	***Prunussubhirtella***
10b	Hypanthium tabulate or campanulate, base not dilated	***Prunuszhuxiensis***
7b	Inflorescences glabrous	**11**
11a	Sepals reflexed	***Prunuscyclamina***
11b	Sepals straight or spreading	**12**
12a	Leaf blade margin serrulate or biserrate with acuminate to aristate teeth	***Prunusserrulata***
12b	Leaf blade sharply serrate	***Prunusconradinae***

## Supplementary Material

XML Treatment for Prunus zhuxiensis

## ﻿Additional information

### Author ORCIDs

Wen-bin Xu https://orcid.org/0009-0002-2645-0053

Xin-wei Li https://orcid.org/0000-0001-6317-1374

### Data availability

All of the data that support the findings of this study are available in the main text or Supplementary Information, or NCBI (https://www.ncbi.nlm.nih.gov/).

## Supplementary materials

Supplementary material 1 Comparison between *P.zhuxiensis* and *P.serrulata* Data type jpg https://binary.pensoft.net/file/1314707This dataset is made available under the Open Database License (http://opendatacommons.org/licenses/odbl/1.0/). The Open Database License (ODbL) is a license agreement intended to allow users to freely share, modify, and use this Dataset while maintaining this same freedom for others, provided that the original source and author(s) are credited.Qi-liang Gan, Wen-bin Xu, Xin-wei Li

Supplementary material 2 ML consensus tree of Prunussubg.Cerasus Data type jpg https://binary.pensoft.net/file/1314708This dataset is made available under the Open Database License (http://opendatacommons.org/licenses/odbl/1.0/). The Open Database License (ODbL) is a license agreement intended to allow users to freely share, modify, and use this Dataset while maintaining this same freedom for others, provided that the original source and author(s) are credited.Qi-liang Gan, Wen-bin Xu, Xin-wei Li
