# Supplementary figures and images for: ﻿Prunuszhuxiensis (Rosaceae), a new species from Hubei, China
Source: PhytoKeys. 2025 Apr 24;255:203–13. doi: 10.3897/phytokeys.255.142428 (PMC12046342; doi:10.3897/phytokeys.255.142428)

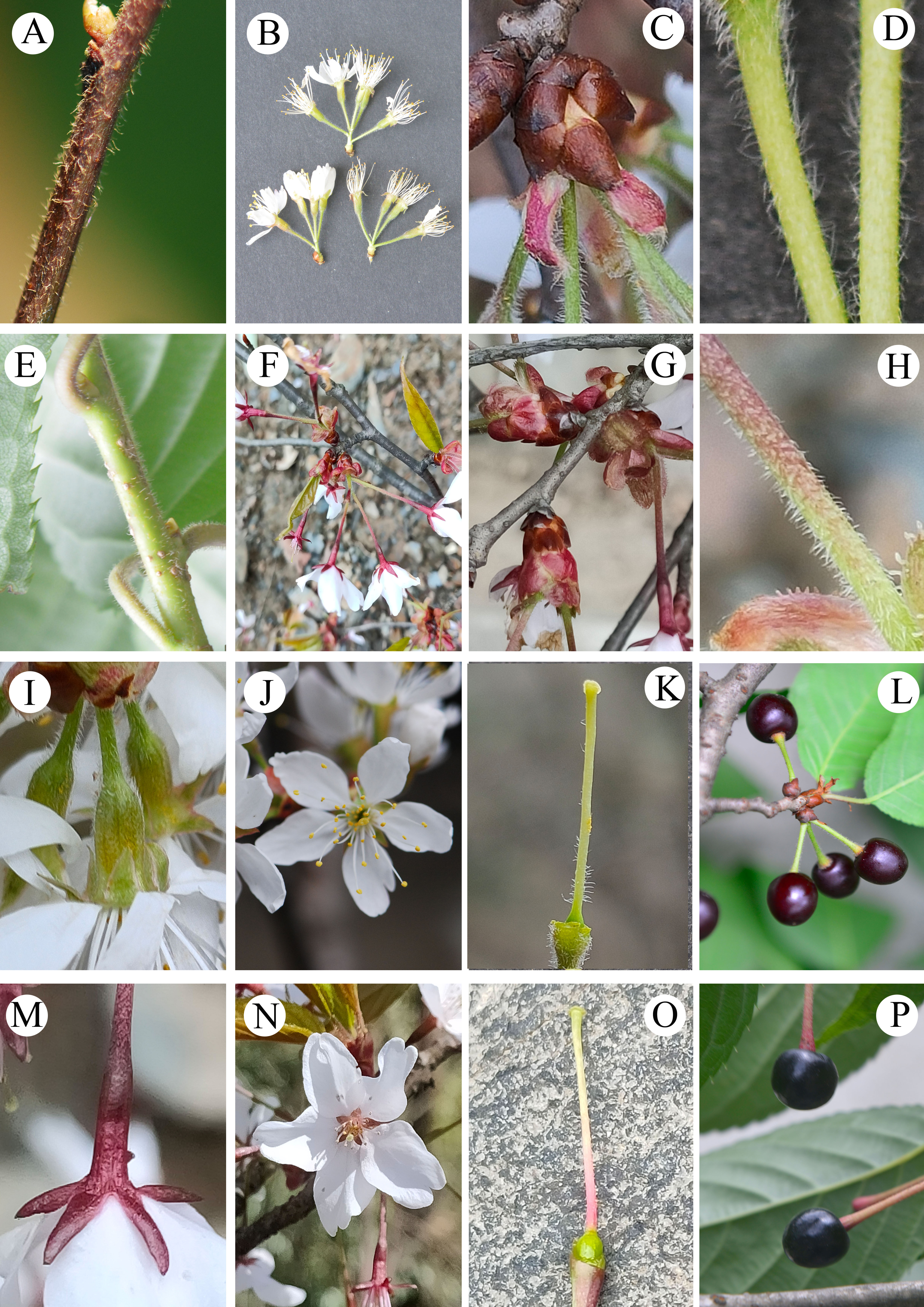

Supplement: Supplementary material 1 — Comparison between P.zhuxiensis and P.serrulata [file phytokeys-255-203_article-142428__-s001.jpg]

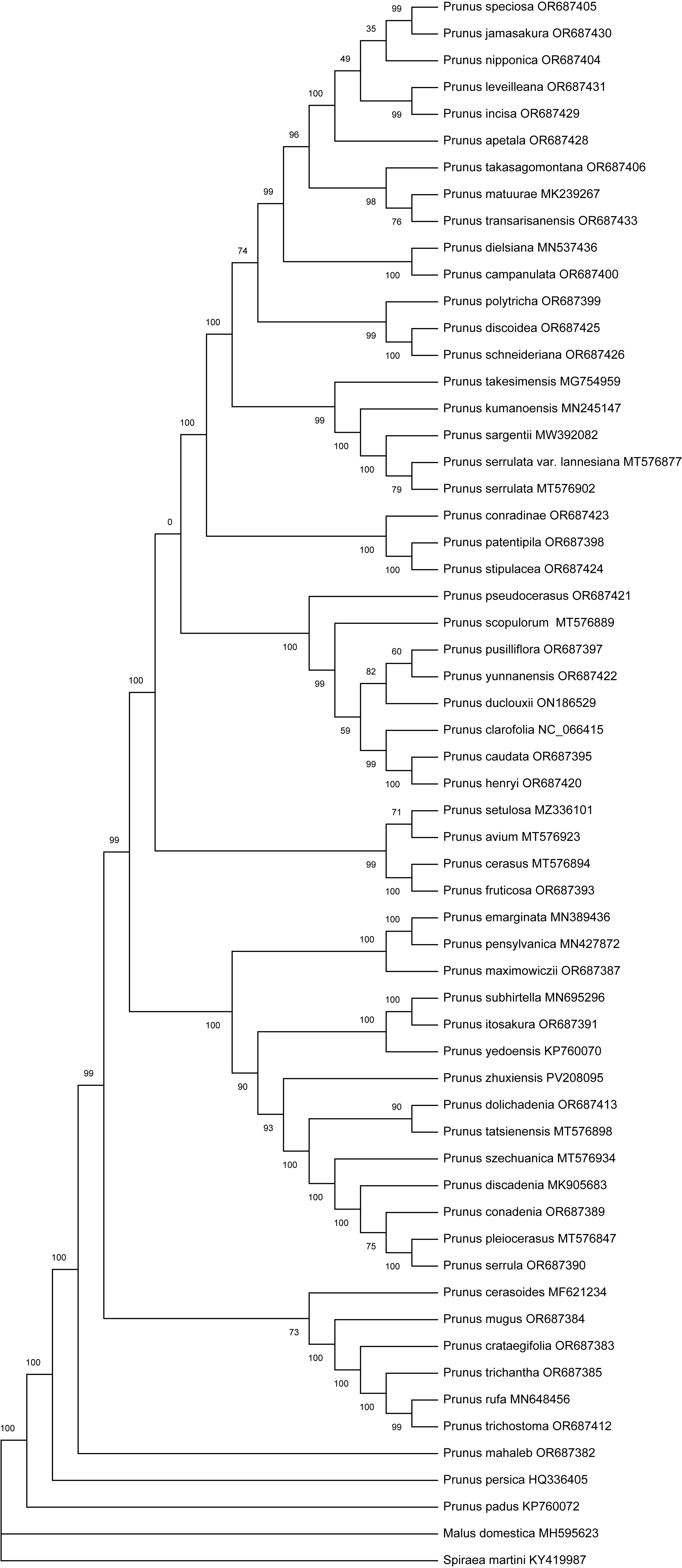

Supplement: Supplementary material 2 — ML consensus tree of Prunussubg.Cerasus [file phytokeys-255-203_article-142428__-s002.jpg]
